# Fluoxetine for motor recovery after acute intracerebral hemorrhage (FMRICH): study protocol for a randomized, double-blind, placebo-controlled, multicenter trial

**DOI:** 10.1186/1745-6215-14-77

**Published:** 2013-03-19

**Authors:** Juan Manuel Marquez-Romero, Antonio Arauz, José Luis Ruiz-Sandoval, Erick de la Cruz-Estrada, Maria Raquel Huerta-Franco, Gerónimo Aguayo-Leytte, Angélica Ruiz-Franco, Humberto Silos

**Affiliations:** 1 Centro de Ciencias de la Salud, Universidad Autónoma de Aguascalientes, Av. Universidad # 940, Ciudad Universitaria, Aguascalientes, 20131, Mexico; 2 Stroke Clinic, Instituto Nacional de Neurología y Neurocirugía “MVS”, Av. Insurgentes Sur # 3877, Col. La Fama, Mexico City, 14269, Mexico; 3 Hospital Civil de Guadalajara “Fray Antonio Alcalde”. Hospital 278, Col. El Retiro, Torre de Especialidades, 8vo. piso, Universidad de Guadalajara Guadalajara, Jalisco, 44280, Mexico; 4 Hospital De Alta Especialidad “Dr. Juan Graham Casasus”. Carretera a la Isla SN. Col. Miguel Hidalgo, Villahermosa, Tabasco, Mexico; 5 Department of Applied Sciences to Work, Health Science Division, University of Guanajuato, 37320 Leon, Guanajuato, Mexico; 6 Centenario Hospital “Miguel Hidalgo”, Galeana Sur 465. Col. Obraje, Aguascalientes, Mexico

**Keywords:** Intracerebral hemorrhage, Stroke, Motor recovery, Fluoxetine, Randomized controlled trial

## Abstract

**Background:**

Spontaneous, nontraumatic intracerebral hemorrhage (ICH) is a subtype of stroke that causes a great amount of disability and economic and social burden. This is particularly true in developing countries where it accounts for between 20% and 50% of all strokes. Pharmacological and surgical interventions have been attempted to reduce the mortality and disability caused by ICH, with unsuccessful results. Recently, the use of fluoxetine in addition to physical rehabilitation has been proven useful to improve motor recovery following cerebral infarct. The purpose of this study is to test whether a 3-month treatment with fluoxetine enhances motor recovery in nondepressed patients with acute intracerebral hemorrhage.

**Methods/design:**

Our study is a randomized, double-blind, placebo-controlled, multicenter clinical trial. We will recruit 86 patients with intracerebral hemorrhage of both sexes, aged >18 years, from four Mexican hospitals. The patients will receive either 20 mg of fluoxetine or a placebo once daily for 90 days. The primary outcome is the mean change in the Fugl-Meyer Motor Scale score between inclusion (day 0) and day 90. The secondary outcomes will be changes in the Barthel Index, the Modified Rankin scale and the National Institutes of Health stroke scale. The outcomes will be measured at day 42 ± 7days and at day 90, for a total of four visits with each subject (at screening and at 0, 42 and 90 days).

**Discussion:**

Current guidelines recommend early supported hospital discharge and home-based rehabilitation programs as the only cost-effective intervention to aid the recovery of patients with intracerebral hemorrhage. Nevertheless, such interventions are dependent on available resources and funding, which make them very difficult to implement in developing countries. We believe that the identification of a helpful pharmacological intervention to aid the motor recovery of these patients will constitute a breakthrough that will have a major impact in reducing the burden of disease caused by this subtype of stroke worldwide, especially in the developing world.

**Trial registration:**

Current Controlled Trials NCT01737541

## Background

Spontaneous, nontraumatic intracerebral hemorrhage (ICH) is a significant cause of morbidity and mortality worldwide, and no treatment has been proven effective [1]. Each year, approximately 1 million people suffer from ICH [2], which causes a great amount of disability and economic and social burden. This is particularly true in developing countries where ICH accounts for between 20% and 50% of all strokes [3]. Hispanic Americans and especially Mexican Americans have been shown to have an increased risk of ICH compared with non-Hispanic whites [4]. The higher prevalence of ICH appears to be very similar in Hispanic patients living in Mexico and in the USA [5]. Despite these data, it is not clear whether this excess in incidence is due to differences inherent to ethnicity, or due to other differences in the disease pathogenesis.

Several therapeutic approaches have been attempted to reduce the burden in terms of death and disability from ICH, including pharmacological and surgical interventions, but trials have been largely unsuccessful [6]. These failures have encouraged a focus on management in intensive care units and physical rehabilitation as the mainstream approaches to reduce mortality and disability, respectively [7].

Recently, the use of antidepressant drugs as an adjunctive treatment to augment recovery in stroke patients has been studied in small clinical trials [8-11]. In 2011, Chollet and colleagues [12] tested adjunctive treatment with fluoxetine in addition to physical rehabilitation to improve motor recovery from cerebral infarct (CI), with excellent results. Subsequent evidence indicated that this improvement is persistent through time [13].

It is thus necessary to elucidate the relative importance and potential benefits of the use of fluoxetine in ICH patients, so that this drug can play a role as an adjunctive therapy to aid the motor recovery of ICH patients, similar to CI patients.

This study will be the first reported randomized, double-blind, placebo-controlled clinical trial of the use of fluoxetine for motor recovery in patients with ICH. The main purpose of this study is to test whether a three-month treatment with fluoxetine enhances motor recovery in nondepressed patients with acute intracerebral hemorrhage.

## Methods/design

### Objectives and hypothesis

#### Objectives

The main objectives of this trial are to (1) compare the magnitude of motor recovery, measured with the Fugl-Meyer Motor Scale (FMMS), in patients receiving fluoxetine with that of patients receiving placebo, and (2) establish the relationship between motor recovery and functional recovery as measured with the Barthel index (BI), the modified Rankin scale and the National Institutes of Health stroke scale (NIHSS).

#### Hypothesis

We hypothesize that the mean change in FMMS between inclusion (day 0) and day 90 will be significantly higher in the patients receiving fluoxetine compared with those receiving placebo.

#### Study design and period

**Figure 1:**
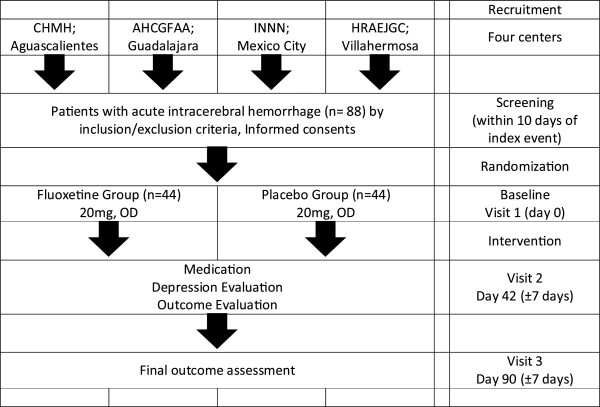
Flowchart of the fluoxetine for motor recovery after acute intracerebral hemorrhage (FMRICH) clinical trial.

This study is a 12-week randomized, double-blind, placebo-controlled, multicenter trial at four Mexican hospitals. Figure 1 shows the schematic flow of the study.

#### Study groups

The study will include the following two arms: the fluoxetine group (treatment arm) and the placebo group (control arm).

### Population

The subjects will be patients of both sexes with acute ICH and ages above 18 years.

#### Eligibility criteria

Inclusion Criteria

1. Men and women ages ≥ 18 years

2. Individuals who meet one of the following criteria:

2.1.  Patients who had an acute intracerebral hemorrhage within the past 10 days causing hemiparesis or hemiplegia

2.2.  Fugl-Meyer motor scale (FMMS) scores ≤ 55

3. Written, informed consent for participation in the trial

Exclusion Criteria

1. Severe post-stroke disability (National Institutes of Health stroke scale [NIHSS] score >20)

2. Premorbid disability, evidenced by residual motor deficit from a previous stroke

3. Comprehension deficits or severe aphasia

4. Previous diagnosis of depression or one of the following:

4.1.  Hospital anxiety and depression scale (HAD) score ≥11 points

4.2.  Taking antidepressant drugs two weeks before inclusion

5. Taking neuroleptic drugs or benzodiazepines 2 weeks before inclusion

6. Other major diseases with life expectancy ≤ 3 months.

Withdrawal Criteria

1. Detection of eligibility violations

2. Poor compliance (<90%) or noncompliance

3. Use of any medication or treatment during the trial that could affect the study results

4. Occurrence of a serious adverse event

4.1. Subject has an acute reaction (allergy, shock) to the investigational product

4.2. Subject develops depression, evidenced by HAD score ≥11 points at Visit 2

5. Subject withdraws consent or is uncooperative

The patients withdrawn after randomization will be followed for outcomes

#### Interventions

Participating subjects assigned to either the treatment group or the placebo group will be instructed to take a pill of fluoxetine (20 mg) or placebo once daily for 90 days. Fluoxetine and placebo tablets will be identical in form, color, odor and packaging.

#### Concomitant treatments and forbidden drugs

The product name, use, dosage and duration of all medications used by the patients during their hospital stay and follow-up will be recorded. Only the use of antidepressants will be prohibited.

#### Sample size calculation

A sample size of 35 in each group will be sufficient to detect a clinically important difference of 14.5 points on the FMMS, assuming a standard deviation of 21.3 FMMS points on the basis of the findings of the fluoxetine in motor recovery of patients with acute ischaemic stroke (FLAME) trial [12], using a two tailed *t*-test of the difference between means, a power of 80%, and a significance level of 5%. This number has been increased to 44 per group (total of 88), to allow for a predicted 20% loss to follow-up.

The formula for the sample size for comparison of two means (two-sided) is as follows:

$$n=\frac{2\sigma^{2}{\left(Z_{a}+Z_{b}\right)}^{2}}{\delta^{2}}$$

$$Z_{a}=1.96$$

$$Z_{b}=0.84$$

δ = size of difference of clinical importance = 14.5

σ = standard deviation of the primary outcome variable = 21.3

$$n=\frac{2*21.3^{2}{\left(1.96+0.84\right)}^{2}}{14.5^{2}}\approx 35$$

Adjusted for 20% patients lost to follow-up:

$$n_{c}=\frac{n}{1-l}\approx 44$$

*l* = expected frequency of patients lost to follow-up = 0.20

#### Randomization method

A pharmaceutical laboratory (Psicofarma™ S.A. de C.V.) will be responsible for the manufacture and randomization of the investigational product, which will be achieved using a web-based randomization program. This program will be set to assign participants equally to each site at a ratio of 1:1.

Each of the sites will be assigned 22 participants. The manufacturer will then deliver the pre-randomized bottles containing the investigational product to each recruiting center.

Study subjects who satisfy the eligibility criteria at each recruiting center will receive the investigational product corresponding to a consecutive number assigned according to their entrance to the study.

#### Blinding

Both the investigator and the subject will be blinded to the assignment of the study drugs. The manufacturer of the tablets will label the investigational drugs by the randomization code number. The labeled experimental products will be provided to the recruiting centers by the manufacturer. An envelope containing all randomization codes will be delivered to the principal investigator and will be kept sealed until the conclusion of the trial.

### Recruitment

Participants will be recruited at the emergency departments at four Mexican hospitals:

The Centenario Hospital ‘Miguel Hidalgo’ (located in Aguascalientes, a midsized city in central México), the Instituto Nacional de Neurología y Neurocirugía (located in Mexico City), the Antiguo Hospital Civil de Guadalajara ‘Fray Antonio Alcalde’ (located in Guadalajara, a major city in western Mexico) and the Hospital Regional de Alta Especialidad ‘Dr. Juan Graham Casassus’ (located in Villahermosa, a midsized city in the south of Mexico).

#### Study schedule

The measurements that will be carried out at each visit are listed in Table 1.

**Table 1:** Study schedule of fluoxetine for motor recovery after acute intracerebral hemorrhage (FMRICH) clinical trial

	**Screening**	**Visit 1**	**Visit 2**	**Visit 3**
Informed consent	•			
Inclusion criteria	•			
Demographics^a^	•			
Inclusion/exclusion criteria check	•			
Vital signs^b^		•	•	•
Medical/drug use history^c^		•		
Smoking/drinking status		•		
Laboratory tests^d^		•		
Lipid test^e^		•		
Coagulation tests^f^		•		•
Date of bleeding		•		
Condition associated with bleeding^g^		•		
BCT	•			
Date of BCT	•			
Volume of the hemorrhage^h^	•			
Localization of the hemorrhage	•			
Concomitant medication	•	•	•	•
Adverse event			•	•
NHISS		•	•	•
Depression Scale	•		•	•
Fugl-Meyer Motor Scale	•		•	•
Patient’s rehabilitation log			•	•
Barthel Index		•	•	•
Modified Rankin Scale		•	•	•
Pill count			•	•

^a^Age, gender, educational level, marital status, occupational status, date of birth, contact address and telephone number. ^b^Blood pressure (mmHg), pulse (beats/minute) and body temperature (°C). ^c^Diabetes Mellitus, hypertension, dyslipidemia. ^d^Hemoglobin, hematocrit, red blood cell count, white blood cell count, platelet count, blood urea, nitrogen/creatinine ratio, fasting plasma glucose. ^e^Total cholesterol (mg/dl), high-density lipoprotein cholesterol (mg/dl), low-density lipoprotein cholesterol (mg/dl), very low-density lipoprotein cholesterol (mg/dl), triglyceride (mg/dl). ^f^International Normalized Ratio, prothrombin time, activated partial thromboplastic time. ^g^Trauma, Drugs, bleeding disorder, exercise. ^h^ABC/2 method. BCT, Brain Computed Tomography; NIHSS, National Institutes of Health Stroke Scale.

### Measurement tools

#### Questionnaires

##### The fugl-meyer assessment (FMA)

The Fugl-Meyer assessment (FMA) [14] is a stroke-specific, performance-based impairment index. It is designed to assess motor functioning, balance, sensation and joint functioning in hemiplegic post-stroke patients. Each of the five FMA domains can be separated to test a specific construct. The motor domain (FMMS) includes items assessing movement, coordination and reflex action of the shoulder, elbow, forearm, wrist, hand, hip, knee, and ankle. We will use the FMMS to assess the motor recuperation of patients in this study.

### The Barthel index

The Barthel index [15] is a widely used measure of functional disability. The index was developed for use in the rehabilitation of patients with stroke. This index measures the extent to which someone can function independently and has the mobility necessary for their daily life activities. We will use the BI to establish the relationship between motor recovery (FMMS) and functional recovery.

### The hospital anxiety and depression scale

The hospital anxiety and depression scale (HAD) [16] is a scale widely used to evaluate anxiety and depression in hospitalized patients and has been validated in Spanish [17]. It consists of 14 items: the odd items represent the anxiety subscale and the even items the depression subscale, and both are scored between 0 and 3. The total score is obtained by the summation of all items in each subscale. The severity of the condition increases with the score. In a validation study by Abent *et al*. [18], the use of the HAD scale resulted in a sensitivity of no less than 91.7 (specificity: 65.3) using the optimum threshold score of 11. The authors concluded that although there were no substantial differences in the performances of the four depression rating scales that they studied, the HAD scale was preferred in screening for post-stroke depression because it requires the least amount of time to administer. Thus, we will use the HAD to rule out depression in the patients enrolling and participating in this study.

### Compliance

All subjects will be asked to return the remaining tablets at their next visit. The rate of compliance (percentage) will be calculated on the basis of the returned tablets.

Compliance (%) = 100 - returned tablets/expected intake × 100.

Investigational drugs will be distributed in packs of 50 at each visit.

50 = 1 times/day × 7 days/week × 6 weeks/visit + 8 extras.

### Outcomes

Both primary and secondary end points will be measured at each visit according to the study schedule (Table 1).

### Primary outcome

The primary outcome is the mean change in FMMS score between inclusion (day 0) and day 90.

### Secondary outcomes

Both within-group and between-group analyses will be performed for each outcome. The differences in the following variables between the baseline (visit 1) and the last visit (visit 3) will be calculated.

1. Barthel Index

2. Modified Rankin scale

3. NIHSS

### Statistical analysis

#### Efficacy assessment

The primary and secondary outcomes will be analyzed using the intention to treat (ITT) method. The full analysis set for the ITT method will include all randomized subjects, regardless of their subsequent withdrawal after enrollment. Continuous variables will be reported as the means ± SD, and categorical variables will be reported as percentages. The Kolmogorov-Smirnoff test will be used to test normality for the continuous variables, and baseline characteristics will be compared by either Student’s *t*-test/U Mann-Whitney for continuous variables or the χ^2^ test (Fisher’s exact test when the expected value is <5) for categorical data.

The primary analysis for efficacy (full-analysis set) will consist of a comparison of the change in FMMS scores at 90 days. To handle the missing data derived from withdrawal and lost to follow-up patients, the last available outcome measure value obtained from the withdrawn/lost to follow-up patient will be used for the analysis (last observation carried forward (LOCF) strategy). A multiple linear regression analysis will be performed to control for baseline factors that show an imbalance.

For the within-group analyses, primary and secondary outcome variables will be evaluated by a paired t-test. Alternatively, for non-normally distributed data, a Wilcoxon test will be performed. Analysis of covariance will be used to analyze differences in each group, adjusting for age and NIHSS as covariates. Statistical significance will be defined as P < 0.05. SPSS for Windows version 17.0 software (SPSS, Inc, Chicago, IL, USA) will be used for the analyses.

### Adverse event reporting

Adverse events (AEs) will be recorded in medical diagnostic terminology. Detailed symptoms, duration, severity, causal relationships, actions taken, results and other information will be recorded for each AE. All AEs must be observed and recorded in the case report file (CRF) in the AE report section.

### Data quality control, data collection and data management

Data quality control will be achieved through monitoring during the trial. After checking the written CRF, well-trained clinical research associates of the principal investigator will collect the data.

### Ethical issues

This study has been approved by the institutional review boards (IRBs) at the Centenario Hospital ‘Miguel Hidalgo’, the Antiguo Hospital Civil de Guadalajara ‘Fray Antonio Alcalde’ and the Hospital Regional de Alta Especialidad ‘Dr. Juan Graham Casassus’, and by the Bioethics Committee (reference: Oficio No. CB/304/11) and the Scientific Committee (reference: Oficio No. DIC/547/11) of the Instituto Nacional de Neurología y Neurocirugía.

Written informed consent will be obtained from each individual prior to enrollment. Research will be performed in compliance with the Helsinki Declaration and with the Good Clinical Practice Guidelines.

## Discussion

The degree of recovery and the speed of recovery from disabilities secondary to ICH are different from the recovery of patients with CI, although this difference has rarely been documented [19,20]. This is partly because the evidence comes from countries where there are a disproportionately lower number of patients with ICH compared with CI available to extract adequate conclusions. However, the existing evidence appears to demonstrate that patients with ICH recover faster and to a quantitatively greater degree compared with patients with CI. For example, in the study by Katrak et al. [21], even though the studied patients with ICH had a greater level of disability at discharge, they achieved significantly greater gains in function than patients with CI after rehabilitation. This was the case regardless of the severity of disability on admission.

The study by Nannetti et al. [22], in which 20% of the studied stroke patients suffered from ICH, patients receiving antidepressants (mostly SSRIs) made the most rapid recoveries during the hospital stay period. However, lower levels of improvement were seen after discharge, which the authors attribute to the fact that the antidepressant treatment was not continued after discharge by some patients (20.4%). This recovery was observed in the group of patients with post stroke depression (PSD) as well as in those without PSD.

The aforementioned data indicate there is a greater amount of potential motor recovery for patients with ICH compared with those with CI. Still, approximately half of ICH survivors will remain dependent on others for daily life activities [23]. The motor recovery mechanisms after stroke are still being elucidated, and most of the studies have focused on patients with CI, so little is known about the motor recovery mechanism for patients with ICH [24]. It has been demonstrated that the presence of wallerian degeneration in the pyramidal tract remote from the initial lesion on magnetic resonance (MR) images correlates with a poor outcome both in CI [25] and in ICH [26] and that the primary motor cortex (M1) plays a key role in the motor recovery of stroke patients. Strategies aimed to facilitate activity in the ipsilesional M1 or to downregulate activity in the contralesional M1 have been found to be the most useful [27]. This ipsilesional facilitation/contralesional downregulation has been attempted by modulation of somatosensory input originating in the paretic or healthy hands. Ipsilesional facilitation/contralesional downregulation is also one of the apparent mechanisms of action of selective serotonin re-uptake inhibitors (SSRIs) in the motor recovery of stroke patients, in addition to cortical reorganization and changes in the somatotopy of M1 [28].

Current guidelines recommend early supported hospital discharge and home-based rehabilitation programs as the only cost-effective intervention to aid in the recovery of ICH patients [1]. Such interventions are highly dependent on available resources and funding, which make them very difficult to implement in developing countries.

In Mexico, ICH accounts for nearly 30% of all stroke cases and produces significant functional disability and death, with 30% mortality and another 30% of patients severely disabled (modified Rankin scale >3) [29]. Up to 25% of Mexican family caregivers have to stop or cut back on work to care for stroke survivors [30]. We strongly believe that identification of a pharmacological intervention to aid in the motor recovery of patients with ICH will constitute a breakthrough that will have a major impact on the burden of disease derived from ICH in our country. We also theorize that the effect size should be bigger than that found in patients with CI.

### Trial status

The trial was first designed in 2011, and subject recruitment began in November 2012.

## Abbreviations

AE: adverse event; AHCGFAA: Antiguo Hospital Civil de Guadalajara ‘Fray Antonio Alcalde’; BCT: brain computed tomography; BI: Barthel index; CHMH: Centenario Hospital ‘Miguel Hidalgo’; CI: cerebral infarct; CRF: case report file; FLAME: fluoxetine in motor recovery of patients with acute ischaemic stroke; FMA: Fugl-Meyer assessment; FMMS: Fugl-Meyer motor scale; HAD: hospital anxiety and depression scale; HRAEJGC: Hospital Regional de Alta Especialidad ‘Dr. Juan Graham Casassus’; ICH: intracerebral hemorrhage; ITT: intention-to-treat; INNN: Instituto Nacional de Neurología y Neurocirugía; LOCF: last observation carried forward; M1: primary motor cortex; MR: magnetic resonance; NIHSS: National Institutes of Health stroke scale; PSD: post-stroke depression; SSRIs: selective serotonin re-uptake inhibitors.

## Competing interests

The authors declare that they have no competing interests.

## Authors’ contributions

JMM is the Chief Investigator for FMRICH and leads the project. AA is the trial director and was responsible for the initial development of the protocol along with JMM. MRH advised on the statistical, sample size and data analysis sections. JMM coordinated the development of trial documentation and ethics approval, and prepared and edited this article for journal publication. JLR, GA, EC, AR, HS are all co-investigators on the project and have made contributions to developing the protocol prior to the start of the trial. All authors have commented on this article. All authors read and approved the final manuscript.
